# Lightweight Algorithm for Apple Detection Based on an Improved YOLOv5 Model

**DOI:** 10.3390/plants12173032

**Published:** 2023-08-23

**Authors:** Yu Sun, Dongwei Zhang, Xindong Guo, Hua Yang

**Affiliations:** 1 College of Information Science and Engineering, Shanxi Agricultural University, Jinzhong 030801, China; sunyu@sxau.edu.cn (Y.S.); run-faster@outlook.com (D.Z.); gxd@sxau.edu.cn (X.G.); 2 College of Computer Science and Technology, North University of China, Taiyuan 030051, China

**Keywords:** YOLOv5, lightweight, attention mechanism, object detection, deep learning

## Abstract

The detection algorithm of the apple-picking robot contains a complex network structure and huge parameter volume, which seriously limits the inference speed. To enable automatic apple picking in complex unstructured environments based on embedded platforms, we propose a lightweight YOLOv5-CS model for apple detection based on YOLOv5n. Firstly, we introduced the lightweight C3-light module to replace C3 to enhance the extraction of spatial features and boots the running speed. Then, we incorporated SimAM, a parameter-free attention module, into the neck layer to improve the model’s accuracy. The results showed that the size and inference speed of YOLOv5-CS were 6.25 MB and 0.014 s, which were $\frac{4}{5}$ and 1.2 times that of the YOLOv5n model, respectively. The number of floating-point operations (FLOPs) were reduced by 15.56%, and the average precision (AP) reached 99.1%. Finally, we conducted extensive experiments, and the results showed that the YOLOv5-CS outperformed mainstream networks in terms of AP, speed, and model size. Thus, our real-time YOLOv5-CS model detects apples in complex orchard environments efficiently and provides technical support for visual recognition systems for intelligent apple-picking devices.

## 1. Introduction

The apple is a significant fruit with both economic and nutritional value. In 2020, according to the statistics of the Food and Agriculture Organization of the United Nations (FAO), the area of apple cultivation in the world was 462 hectares, with a annual global production of 86.44 million tonnes. China is the world’s largest producer and consumer of apples, accounting for over 50% of global cultivation, which shows the importance of the apple industry [1,2]. In traditional apple orchards, fruit picking relies mainly on manual labor and suffers from low efficiency, high cost, and insufficient delivery. The development of automatic apple-picking robots is of great significance. It is essential to employ computer vision for the rapid detection, identification, and precise location of the apple fruit in automated picking [3,4,5].

With the advances in artificial intelligence, machine learning methods have been widely applied in computer vision tasks. Machine learning mainly uses features that can provide robust representation, such as color space conversion, histograms of oriented gradients (HOG) [6], and Haar-like [7] features. Based on extracted features, the researchers identified the object by using the threshold segmentation method [8], color difference method [9], K-means clustering algorithm [10], region growing method [11], support vector machine (SVM) [12], k-nearest neighbor method (KNN) [13], and a combination of multiple algorithms. Bulanon et al. [8] used the optimal threshold segmentation with intensity histogram and maximum grey level to classify the fruit and the background. Lak et al. [10] utilized edge detection and a combination of color and shape analysis to segment images of red apples. Peng et al. [14] used the Otsu algorithm to segment the fruit image and the Canny edge operator to extract the edges of the fruit. They then applied SVM to classify the six fruits.

However, machine learning methods can only be adapted to specific conditions. Detecting fruit bodies in complex orchard environments such as branch occlusion, fruit overlap, and illumination variations remains a challenge. The detection accuracy of machine learning methods cannot meet the requirements of agricultural production.

With the developments in deep learning, more powerful tools are introduced for tasks such as image classification, object detection, segmentation, and registration. Various networks, such as Visual Geometry Group (VGG) [15], AlexNet [16], and Residual Net (ResNet) [17], have been extensively studied to improve the model performance. Loui et al. [18] applied deep convolutional neural network (CNN) to identifying tomato leaf disease With AlexNet, GoogLeNet, and ResNet as the backbone, respectively. The result shows that CNN based on the optimal model ResNet with stochastic gradient descent (SGD) achieved the highest accuracy of 97.28%. Guan et al. [19] trained a series of deep convolutional neural networks to diagnose disease severity. The results show that the deep VGG16 model trained with transfer learning yields an overall accuracy of 90.4%.

Object detections [8,20,21] based on convolutional neural networks (CNNs) fall into two categories, and one is a two-stage proposal-driven approach: the first stage generates candidate object locations; the second stage classifies the locations. Recent works on two-stage detectors, such as R-NN [22], Faster R-CNN [23], and R-FCN [24] models, have high detection accuracy and strong generalization ability. GAO et al. [25] used the improved Faster R-CNN network to detect apples in dense-leaf trees. The mAP was 87.9%, and the average detection time per image was 0.241 s. However, this model does not meet the real-time requirements due to its low running speed and large model size. In contrast, the other option is a one-stage detector that predicts the classification of objects over regular dense proposals. Detectors working on one-stage methods are represented by SSD [26], YOLO [27], and RetinaNet [28], which are more attractive due to their fast running speed and simple architecture. One-stage neural networks are widely used in the manufacturing industry, medicine, and agriculture. Liu et al. [29] developed a YOLOv3 model with four-scale detection layers (FDL) and ground-penetrating radar (GPR) to detect pavement cracks for transportation infrastructure assessment. Sha et al. [30] developed fine-tuned YOLOv5 models to detect the quality of solder joints on aviation plugs. Experimental results in the actual production line show that the method achieves a detection accuracy of more than 97.5%, with a detection speed of about 0.1 s.

Convolutional neural networks have also been used to improve the accuracy and robustness of apple-picking robots [31,32]. Sun et al. [33] proposed an improved RetinaNet apple detection network based on the backbone ResNet50 module and the weighted bidirectional feature pyramid network (BiFPN). The mAP is 91.26%, and it takes 42.72 ms to detect an apple image. Tian et al. [34] proposed a YOLOV3-dense model used on apples during different growth stages in orchards with fluctuating illumination, complex backgrounds, overlapping apples, and branches and leaves. The detection time of the model is 0.304 s per frame at 3000 × 300. Zhou et al. [35] proposed an improved YOLOv4 network and a threshold-based bounding box matching merging algorithm to identify apples in panoramic images. This network improves the small object detection accuracy and achieves panoramic image recognition. Wu et al. [36] replaced the backbone network Cross Stage Partial Darknet53 (CSPDarknet53) of the YOLOv4 model with EfficientNet and added a convolution layer (Conv2D) to the three output layers—their EfficientNet-B0-YOLOv4 model achieved a precision of 95.52% and 0.338 s detection time.

However, these models are computationally intensive, time-consuming, and are challenging to generalize in this area. Although the recognition speed of the ordinary lightweight model is significantly improved, the recognition accuracy does not meet the requirements. Mazzia et al. [37] employed a YOLOv3-tiny algorithm on embedded platforms such as Raspberry Pi 3 B+ with Intel Movidius Neural Computing Stick (NCS), Nvidia’s Jetson Nano, and Jetson AGX Xavier. The model was deployed on embedded hardware with 83.64% accuracy and achieved a 30 fps frame rate for complex scenarios. Lv et al. [38] proposed a citrus recognition method based on the improved YOLOv3-LITE lightweight neural network, with an average precision (AP) value of 91.13%. The detection of citrus objects on a GPU can reach 246 FPS, and the inference speed of a single image is 16.9 ms. Zhang et al. [39] proposed a model based on MobileNet V3 backbone and a lightweight attention mechanism combined with improved YOLOv4 to detect potato individuals in different environments, with a detection time of 43 ms and an average accuracy of 91.4%.

Although the YOLO-based network significantly improves the detection accuracy and reduces false and missed detection, the picking robot carries an embedded platform with limited arithmetic resources. The detection speed of complex models cannot meet the real-time demand and is challenging to deploy. We evaluated the recent popular deep learning networks and used YOLOv5 as the baseline. We proposed a lightweight real-time apple detection model, YOLOv5-CS, which uses ripe apples in an natural non-structured orchard environment as the study object. Our contributions are summarized as follows:

- We created a well-labeled dataset of apples. The dataset consists of apple images captured in complex, unstructured environments, with instances of branch occlusion, fruit overlap, and illumination variations.
- Based on YOLOv5n, we introduced the C3-light module in the feature extraction layer to improve the network structure and integrated the SimAM attention module into the feature fusion layer to improve the model accuracy. Finally, we proposed a lightweight real-time YOLOv5-CS model for apple detection.
- We conducted extensive experiments on the apple detection task. Our YOLOv5-CS achieved substantially lower weight size and higher running speed compared to others while maintaining accuracy.

## 2. Results

### 2.1. Performance of YOLOv5-CS

**Figure 1:**
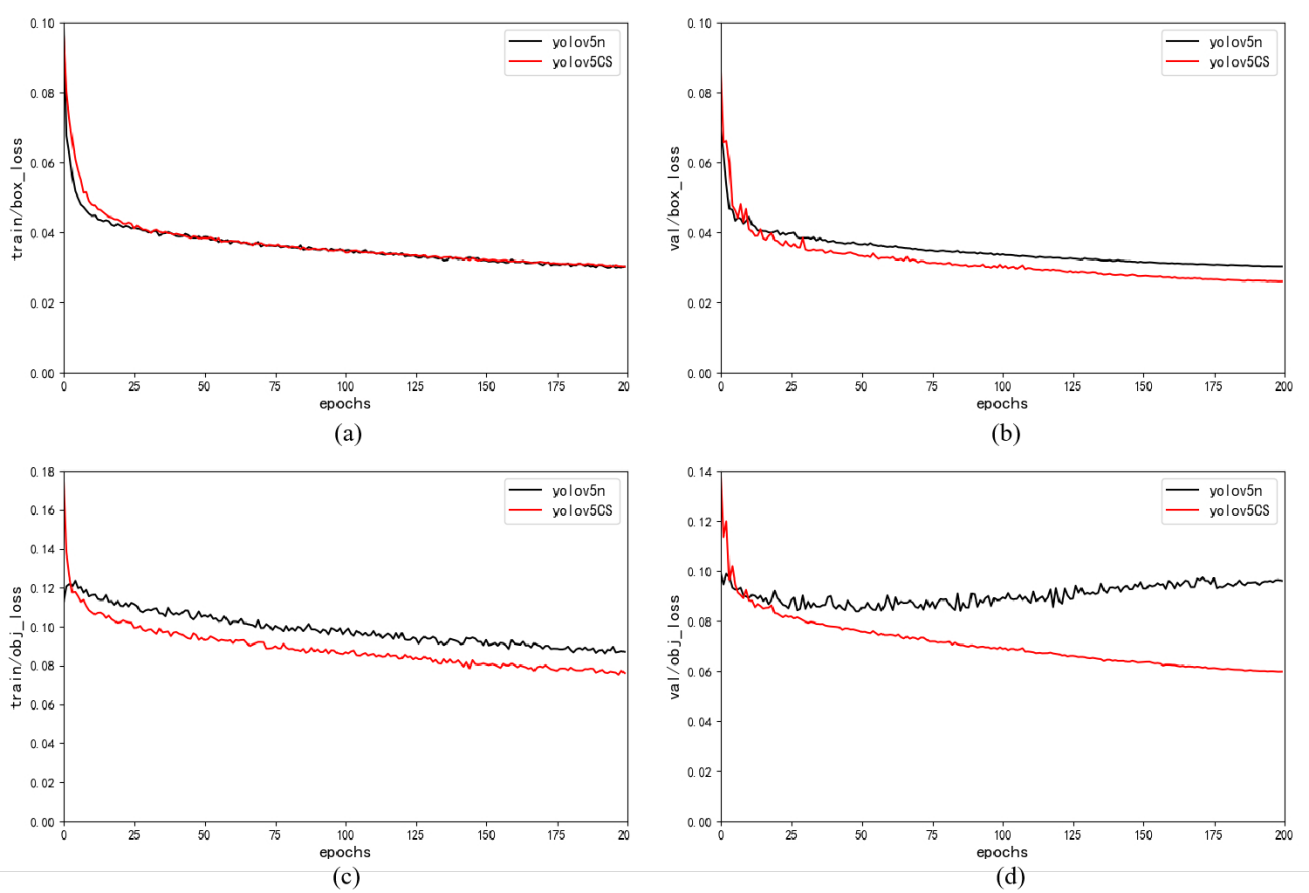
Training loss (**left**) and validation loss (**right**) curves of YOLOv5-CS and YOLOv5n models: (**a**,**b**) box_loss; (**c**,**d**) obj_loss.

We evaluate the performance of the proposed YOLOv5-CS and YOLOv5n on our apple dataset. The comparison curves of the training loss and validation loss are shown in Figure 1.

We can see that the training and validation loss curves of YOLOv5-CS converge faster than those of YOLOv5n and gradually decrease until they stabilize within 200 epochs. This suggests that YOLOv5-CS extracts features more efficiently, accelerates the model’s convergence, and finally runs faster.

**Figure 2:**
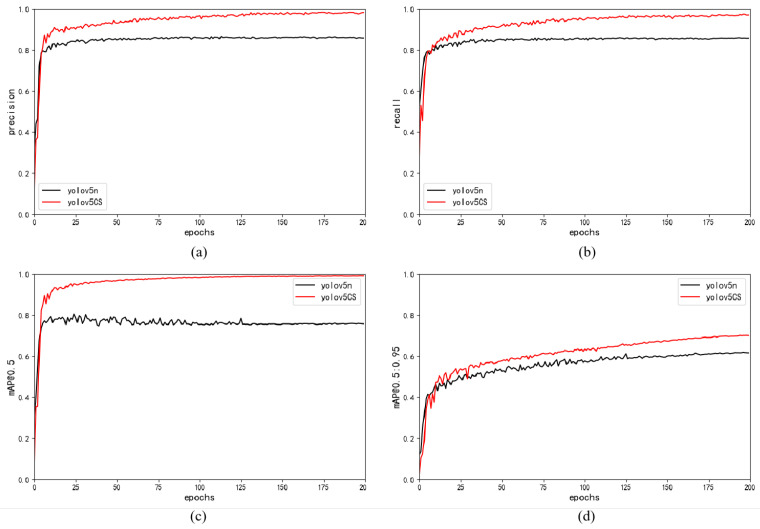
Performance curves of YOLOv5-CS and YOLOv5n model: (**a**) precision; (**b**) recall; (**c**) mAP${}_{0.5}$; (**d**) mAP${}_{0.5:0.95}$.

Figure 2 and Table 1 compare the detection performance of YOLOv5-CS and YOLOv5n. Figure 2 demonstrates that by introducing the C3-light and SimAM modules into YOLOv5n, we substantially improve the network performance. Specifically, our YOLOv5-CS significantly improves precision, recall, mAP${}_{0.5}$, and mAP${}_{0.5:0.95}$ compared to YOLOv5n.

Table 1 demonstrates that our YOLOv5-CS achieves very competitive detection performance, while being efficient in terms of speed and model size. Specifically, the precision, recall, F1, AP, and mAP${}_{0.5:0.95}$ of the YOLOv5-CS model reach 97.81%, 97.32%, 97.55%, 99.10%, and 70.25%, respectively, which is 13.84%, 13.47%, 13.69%, 30.43%, and 13.62% higher than that of YOLOv5n. In addition, the memory size of the YOLOv5-CS model is 6.25 MB, which is approximately $\frac{4}{5}$ of YOLOv5n. The FLOPs of YOLOv5-CS are reduced by 15.56%, and the inference time is 14.1 f/ms (On GPU), i.e., slightly higher than YOLOv5n. Our improvement of YOLOv5n can largely boost the baseline accuracy and performance of apple detection in complex natural environments.

### 2.2. Performance Comparison of Different Models

Table 2 shows the superiority of our YOLOv5-CS over seven popular methods (i.e., YOLOv8, YOLOv7, YOLOv5l, YOLOv5m, YOLOv5s, RetinaNet [28], and Faster R-CNN [23]) on the same apple dataset. YOLOV5-CS consistently outperforms representative models by having the highest efficiency in balancing accuracy, model size, and speed trade-offs on GPU.

Specifically, compared with the recent one-stage method, our YOLOv5-CS is as accurate as YOLOv8, while the model size is about 1/14 of YOLOv8. The FLOPs is reduced by 97.7%, and inference speed is reduced by 60.2% on our desktop computer. YOLOv8 has large backbone networks, and its model size is nearly 83.7 MB, yielding high accuracy and slower inference speeds. Compared with YOLOv7, our YOLOv5-CS achieves a healthy 10.79 point AP gap (99.10% vs. 88.31%) while being simpler and faster.

In addition, We trained the YOLOv5 family (YOLOv5s, YOLOv5m, YOLOv5l) on our dataset. The YOLOv5n network has the smallest depth and width of feature maps in the YOLOv5 family, and the others go deeper and wider on top of that. Compared with YOLOv5s, YOLOv5m, and YOLOv5l, YOLOv5n is the simplest and fastest, while it has the lowest accuracy. To meet the accuracy and trade-off between model size and detection speed, we take YOLOv5n as the base model and improve it. Moreover, the precision of the YOLOv5-CS is 14.33% higher than RetinaNet. The memory size is 1/17 of RetinaNet, and FLOPS is reduced by 2.56%. The detection speed per image is 14.1 f/ms, i.e., about 79 times faster than RetinaNet.

Compared with the two-stage detector, Faster R-CNN, the precision, recall, AP, and mAP${}_{0.5}$ of the YOLOv5-CS model are 24.52%, 19.05%, 14.76%, and 22.91% higher than that of Faster R-CNN, respectively. The YOLOv5-CS model has a memory size 1/84 that of Faster R-CNN, with the FLOPs being reduced by 75.48%, and it is also 64 times faster than Faster R-CNN.

**Figure 3:**
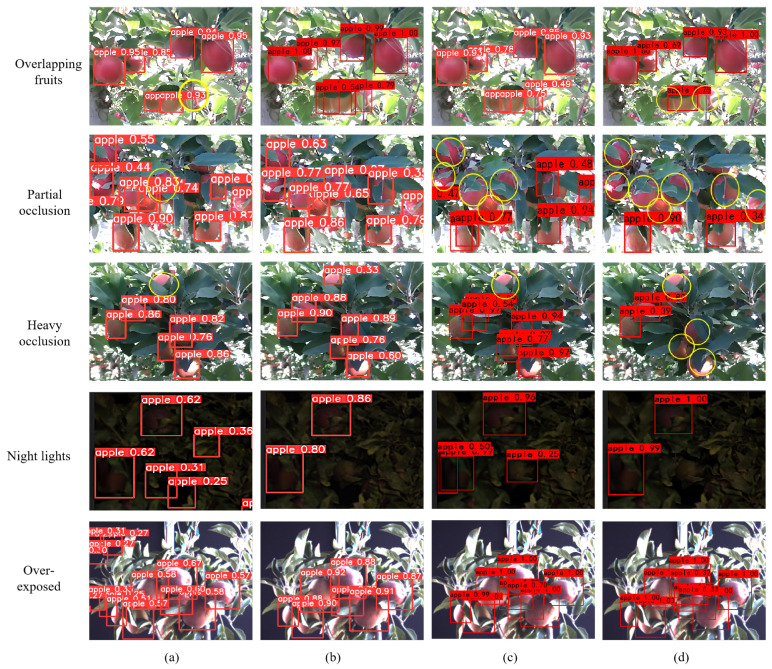
Comparison of detection results with different models: (**a**) YOLOv5; (**b**) YOLOv5-CS; (**c**) FasterR-CNN; (**d**) RetinaNet.

Figure 3 shows the detection results of the four representative models: YOLOv5n, YOLOv5-CS, RetinaNet, and Faster R-CNN, where the red rectangles represent predictions of the model, and we mark the missed objects with yellow circles. We can see that in the overlapping environment, YOLOv5n and RetinaNet miss objects. In the partial occlusion environment, YOLOv5n, Faster R-CNN, and RetinaNet miss one, five, and seven objects, respectively. In the heavy occlusion environment, only YOLOv5-CS detects all apple objects. YOLOv5n and Faster R-CNN have false detection in the dark and exposed environments. At 640 × 640, our YOLOv5-CS runs significantly faster than other detection methods, with comparable performance on the same GPU.

### 2.3. Ablation Experiments

We have introduced the C3-light module and SimAM attention module into the base network. In order to analyze the contribution of each module, here, we conduct a ablation study. The baseline is YOLOv5n. We add the two modules to the baseline model one by one, with the network setting following Section 3.5. The parameters and performance are shown in Table 3 and Table 4. In Table 4, C stands for the C3-light module, S for the SimAM module, YOLOv5-C for the network formed after C3-light replaced C3 in YOLOv5n, and YOLOv5-CS for the network formed after the SimAM was introduced into YOLOv5-C. “✓ means that the model contains this module, while “×” means it does not.

Results in Table 3 show that C3-light is an appealing choice for simple parameters with reduced FLOPs. The FLOPs of C3-light are only $\frac{7}{10}$ that of C3, and the parameters decreased by 31.65%.

As presented in Table 4, compared with the baseline network YOLOv5n, the FLOPs of YOLOv5-C are reduced by 15.56%, and the parameters are reduced by 16.3%. The C3-light module accelerated the training speed of the model but with a simultaneous loss in detection accuracy. We then introduced the SimAM module to optimize the YOLOv5-C. We improved the AP by 28.73% (from 70.63% to 99.10%) under similar FLOPs and parameters. It is not unsurprising because SimAM is a lightweight module designed for detection tasks and adds no parameters to the network, which means the model size has approximately no increase.

## 3. Materials and Methods

### 3.1. Dataset Processing

#### 3.1.1. Dataset

In our study, apple dataset contains dataset A and dataset B. We collected apple images of dataset A in an unstructured apple orchard in Yuci County, Jinzhong City, China. The orchard is located at 36°45${}^{\prime }$ N and 113°6${}^{\prime }$ E. We collected images with a DS-330 camera (FUJIFILM Investment Co.,Ltd., Taiyuan, China) at daytime and at night under a LED light in August 2022. Images, including branch occlusions, fruit overlaps, and lighting variations, were taken at different distances and angles. A total of 200 apple images were obtained in dataset A with a size of 1280 × 960 and a resolution of 96 dpi. We downloaded dataset B from the GitHub repository https://github.com/fu3lab/Scifresh-apple-RGB-images-with-multi-class-label (accessed on 23 March 2023). Dataset B included 300 images collected in a modern farmed apple orchard near Prosser, Washington. The acquisition device was a KinectV2 camera (Microsoft, China), and the resolution of the apples was 1920 × 1080 pixels.

#### 3.1.2. Dataset Labeling

**Figure 4:**
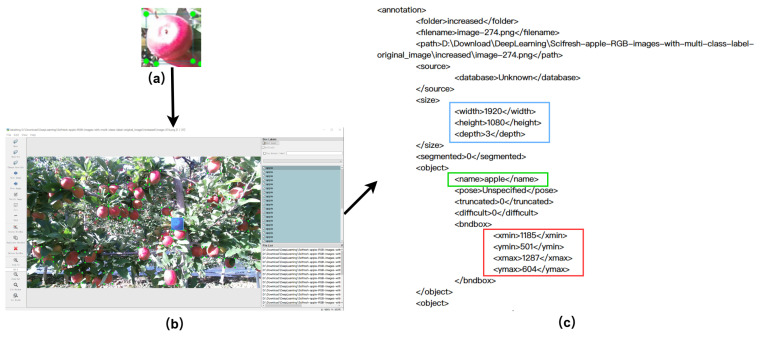
Labeling process: (**a**) a labeled apple; (**b**) annotation of an image; (**c**) annotation file: <size> denotes the length and width of the image and the number of channels; <object> denotes the category of the labeled object (“apple”); <bndbox> denotes the pixel coordinate position of the labeled object, which contains the upper-left and lower-right coordinates of the labeled apple.

We labeled the images of apples with the LabelImg (v1.8.6), and the apples were labeled as “apple”. We obtained a total of 7046 labeled ”apple”. The labeling process is shown in Figure 4. The coordinate position of the labeled object was saved as an .XML annotation file. The file was then normalized as a .TXT file to comply with the YOLO file format.

#### 3.1.3. Dataset Augmentation

Expanding the dataset through the implementation of data augmentation can significantly improve the robustness and generalization of the model and prevent the model from over-fitting. We used the following data augmentation methods:

- The Gaussian blurring and average blurring processes reduce the detail of the image;
- The color space adjustment enhances the detection performance of the model in various gray levels;
- The brightness adjustment improves the detection performance of models under different illumination conditions;
- The horizontal and vertical flip increase the growth directions of branches and apples of the dataset;
- The image zoom increases the amount of small object detection.

**Figure 5:**
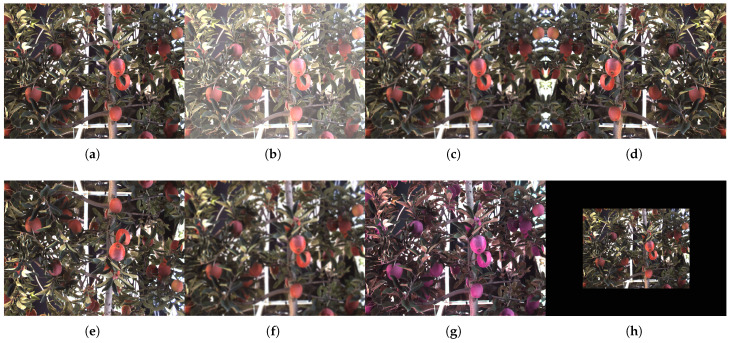
Apple Images of data augmentation: (**a**) original; (**b**) brightness adjustment; (**c**) GaussianBlur; (**d**) flip horizontally; (**e**) flip vertically; (**f**) AverageBlur; (**g**) Colorspace adjustment; and (**h**) Resize.

The parameters of eight data augmentations are shown in Table 5, and the result of the data augmentation is shown in Figure 5.

We obtained a total of 2700 apple images and 7046 labeled “apple” with the implementation of data augmentation. We divided the dataset into three sets: a training set, a validation set, and a test set, and the ratio of images in each set is 8:1:1. We used 2160 images to train the model, including 29,600 “apple” labels; 270 images to select and adjust the parameters of the model to improve the generalization ability of the model and prevent overfitting, including 3691 “apple” labels; and 270 images to evaluate the generalization error of the model, including 3621 “apple” labels. The types and contents of the datasets are listed in Table 6.

### 3.2. YOLOv5 Network Structure

YOLO [40] is the most classical one-stage target detection algorithm suitable for real-time video and image detection. In general, YOLO series models [41] consist of four main components: input, backbone layer, neck layer, and head layer. The backbone of YOLOv1 is mainly inspired by the structure of GoogLeNet, and it uses the Leaky ReLU activation function. The backbone of YOLOv2 is designed based on YOLOv1 and introduces the BN layer to optimize the model’s overall performance in the Darknet-19 network. Darknet-53 is designed as the backbone of YOLOv3, which significantly improves the architecture of YOLOv2. Base on YOLOv3 and inspired by the network structure of CSPNet, the backbone of YOLOv4 combines multiple CSP submodules to design CSPDarknet53 and utilizes the Mish activation function. YOLOv5’s backbone uses the same CSP ideas as those used in YOLOv4. In addition, the Focus structure was present in the initial version of YOLOv5 but was dropped after the sixth version in favor of regular convolution. Based on YOLOv5, the EfficientRep backbone structure is designed in YOLOv6. Its backbone replaces normal convolutions with the RepConv structure. Moreover, YOLOv6 optimizes the SPPF by introducing a more efficient SimSPPF to increase the efficiency of feature reuse. The backbone of YOLOv7 is based on the E-ELAN and MPConv structures of YOLOv5. YOLOv8, on the other hand, introduces the C2f structure with a richer gradient flow and the decoupled head structure. Moreover, Anchor-Based is substituted by Anchor-Free, while TAL (Task Alignment Learning) dynamic matching is adopted. Additionally, DFL (Distribution Focal Loss) is combined with CIoU Loss to enhance the loss function of the regression branch. YOLOv5, as an improved version of the YOLO series, offers more convenience for deploying the apple recognition model in real-world environments. Moreover, it boasts impressive inference speed and recognition accuracy. Depending on the depth and width of the network, YOLOv5 can be classified as YOLOv5n, YOLOv5s, YOLOv5m, and YOLOv5l. The parameters of the YOLOv5 family are shown in Table 7.

**Figure 6:**
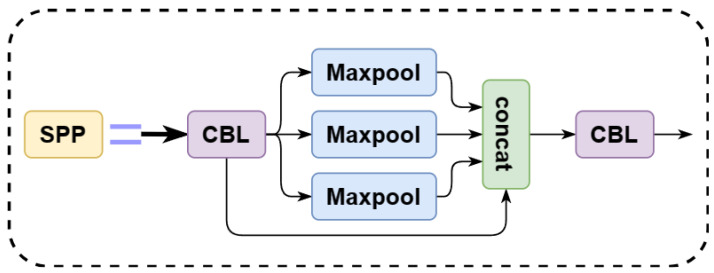
SPP structure.

**Figure 7:**
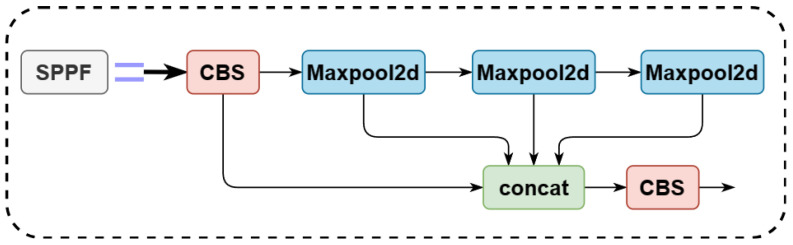
SPPF structure.

**Figure 8:**
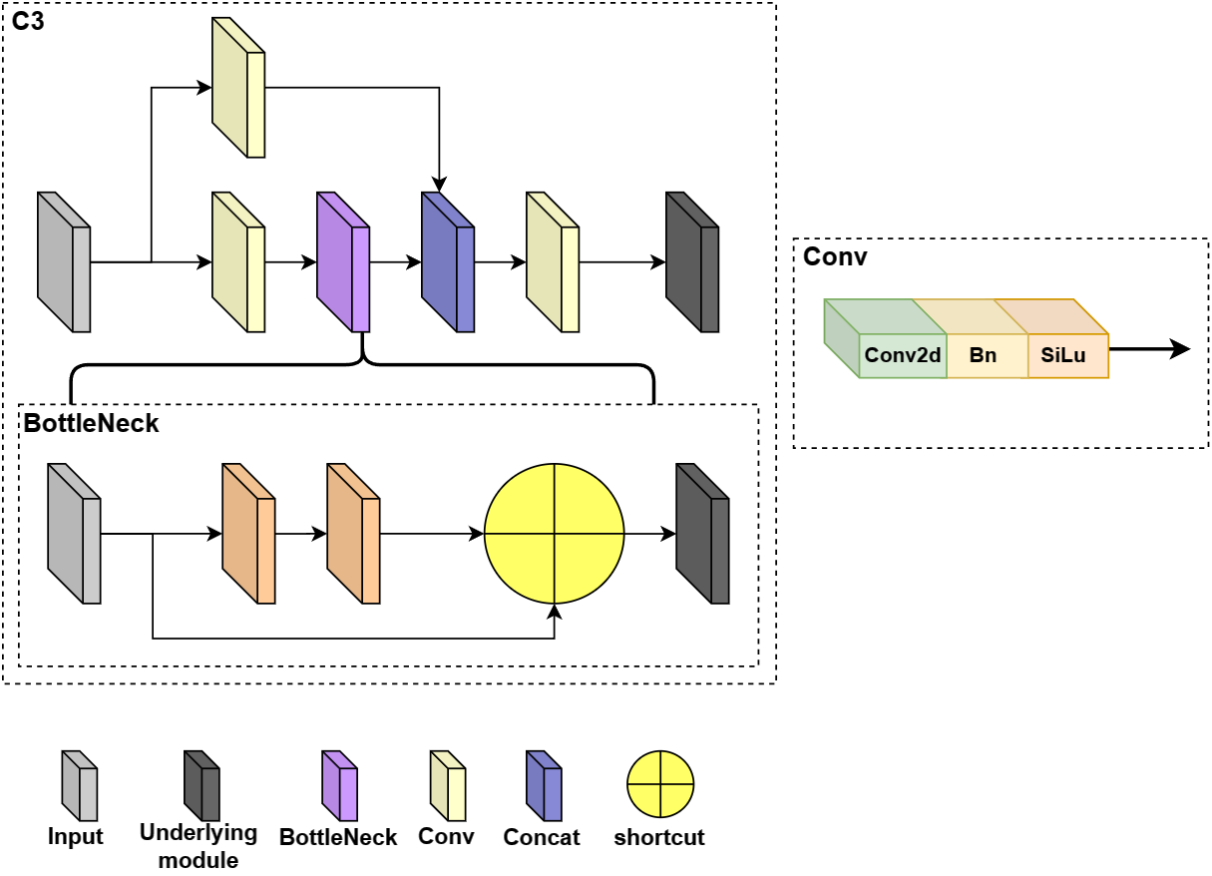
C3 network hierarchy structure.

To balance the performance and inference speed of the recognition task in the orchard environment, we use YOLOv5n as the baseline. YOLOv5n network structure consists of backbone, neck, and head layers [42]. YOLOv5n (v7.0) has three significant improvements over the previous version:

(1) The Focus structure of the backbone layer of the network is changed to a 6 × 6 convolutional layer so that the model can be run more efficiently on the existing GPU devices after optimizing the algorithm.
(2) SPPF replaces SPP in the backbone layer. The inputs in the SPP structure are passed through multiple MaxPool layers of different sizes in parallel and are further fused to solve the target multi-scale problem, as shown in Figure 6. The inputs in the SPPF structure are concatenated through multiple MaxPool layers of size 5 × 5, as shown in Figure 7. SPPF is faster and more efficient while maintaining the same computational results.
(3) In the new CSP-PAN structure of the neck layer, the CSP structure is added to each C3 module to increase the number of channels of the feature map and improve the feature expression ability [43]. The network hierarchy of the C3 structure is shown in Figure 8. The C3 module contains three convolutional layers and multiple Bottleneck modules, where the Bottleneck module consists of two 1 × 1 convolutional layers and one 3 × 3 convolutional layer.

**Figure 9:**
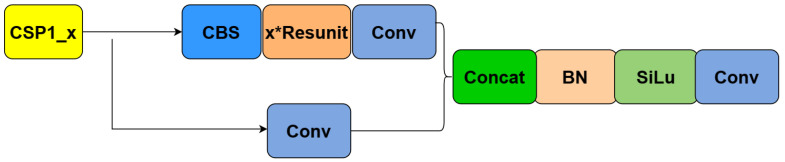
CSP1_x network hierarchy diagram.

**Figure 10:**
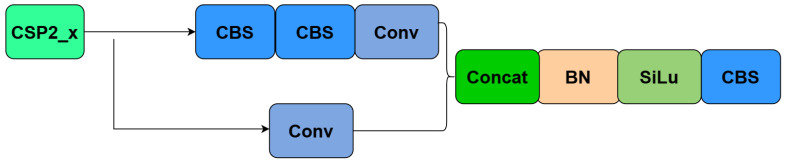
CSP2_x network hierarchy diagram.

There are two designs of CSP structures in YOLOv5, namely, CSP1_X and CSP2_X structures, whose network structures are shown in Figure 9 and Figure 10, respectively. In this way, two branch nodes are introduced on the connected subgraph and these branch nodes are used as nodes in the network topological relation graph to represent the whole network. The CSP1_X structure is applied to the backbone network, which is a deep network. Adding residual structure increases the gradients propagating between layers, avoiding the vanishing of gradients due to deepening, and allowing for more fine-grained features to be extracted without worrying about network degradation. The CSP2_X structure applied to the neck layer can effectively reduce the amount of computational and memory cost and also improve the feature fusion capability of the network to extract more rich, detailed, and semantic information.

### 3.3. Improved YOLOv5-CS Network

**Figure 11:**
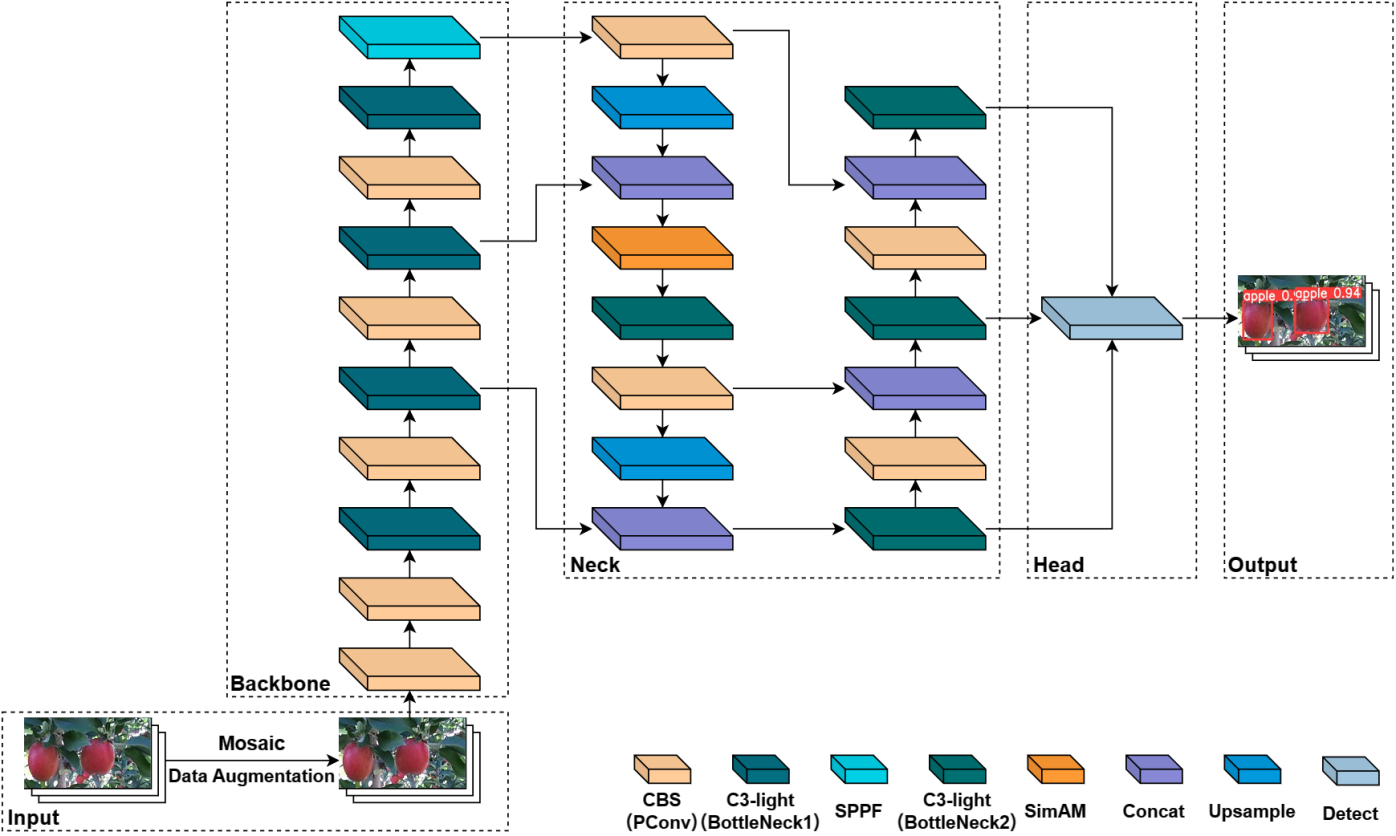
Model structure diagram of improved YOLOv5. We utilize C3-light to replace the C3 module and introduce the SimAM module into the neck layer of the baseline. PConv is partial convolution; SPPF is spatial pyramid pooling layer; SimAM is parameter-free attention coordinate mechanism; Concat is feature splicing layer, the feature map fusion operation; and Upsample is the up-sampling operation.

Based on the YOLOv5n model, we used the lightweight C3-light module to replace the C3 module in the feature extraction layer to improve the lightweight network structure, introduced the parametric-free attention coordinate mechanism SimAM module in the feature fusion layer to improve the model detection accuracy, and proposed the YOLOv5-CS network model. Figure 11 shows the YOLOv5-CS model network structure. We used CIOU loss to calculate the rectangle loss and BCE loss to calculate the confidence loss and classification loss.

#### 3.3.1. The Lightweight C3-Light Module

YOLOv5 achieves excellent recognition accuracy and speed in multi-class detection tasks, but the model has parameter redundancy, consumes additional memory capacity, and has unnecessary computational overhead. To improve the model’s speed, address the problem of low computational power resources of real hardware in practical applications, and meet the requirements of real-time detection, we performed lightweight improvements on its feature extraction network. We lightened the convolutional model by reducing the calculation amount in the convolutional process [44].

**Figure 12:**
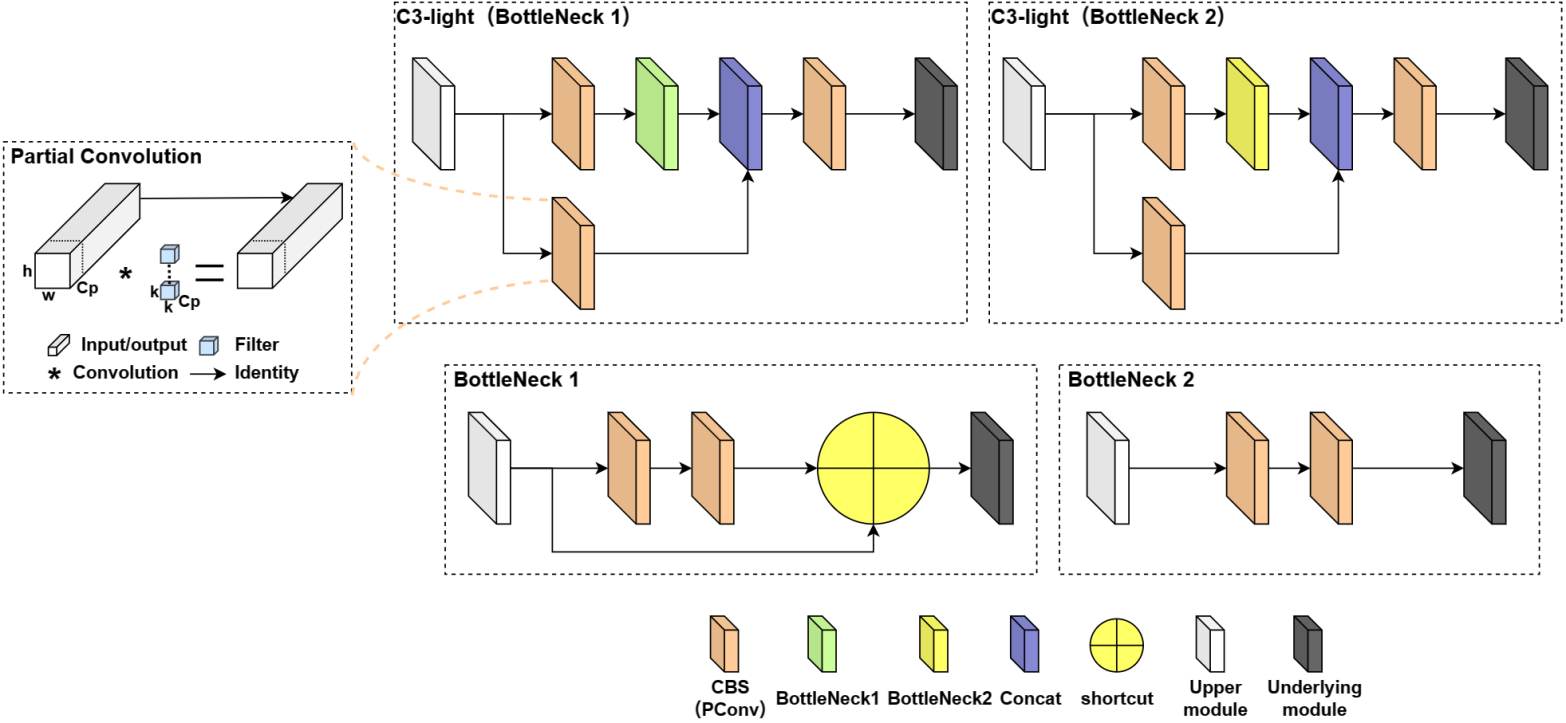
C3-light network structure. For an input I $\in \;R^{c\;\times \;h\times \;w}$, PConv applies $c_{p}$ filter W $\in \;R^{k\;\times \;k}$. This makes PConv have as low Flops as $h\;\times \;w\times \;k^{2}\;\times \;c_{p}^{2}$ compared to a regular Conv with $h\;\times \;w\times \;k^{2}\;\times \;c^{2}$. With a typical partial ratio $\frac{c_{p}}{c}=\frac{1}{4}$, the Flops of PConv are only $\frac{1}{16}$ of a regular Conv.

The C3 module is significant in the YOLOv5 network. Its main objective is to increase the network’s depth and receptive field, enhancing feature extraction ability. In practical applications, we require low latency and high throughput. However, the neural network’s high computational complexity and delay pose challenges. To address this, we utilize partial convolution (PConv) [45] to reduce floating-point operations (FLOPs) and achieve a lightweight network. Specifically, we propose a simple PConv to simultaneously reduce computational redundancy and memory access. Figure 12 illustrates the C3-light network structure and the working mechanism of PConv. PConv applies a regular convolution (Conv) only on a portion of the input channels for spatial feature extraction, leaving the remaining channels unaffected. For consecutive or regular memory access, we consider the first or last consecutive channels as representatives of the entire feature maps for computation. Without loss of generality, we assume that the input and output feature maps have the same number of channels. Based on this, we have designed a C3-light module to replace the C3 module in the original YOLOv5. This modification aims to further lighten the model structure without compromising feature extraction ability. We replaced the C3 modules in the Backbone layer by C3-light (BottleNeck1) and in the Neck layer by C3-light (BottleNeck2).

#### 3.3.2. Feature Fusion Network with the SimAM Attention Mechanism

The accuracy of the YOLOv5n network with the C3-light module decreases when detecting small apples and apples in occluded and dark environments. To enhance the ability of the backbone network to extract global features of apple images, the SimAM module, a simple but effective attention mechanism, is introduced into the model. SimAM [46] is used to enhance the feature expression ability in convolutional neural networks, improving the model’s sensitivity to small and dense apple targets to improve the detection accuracy of the model.

**Figure 13:**
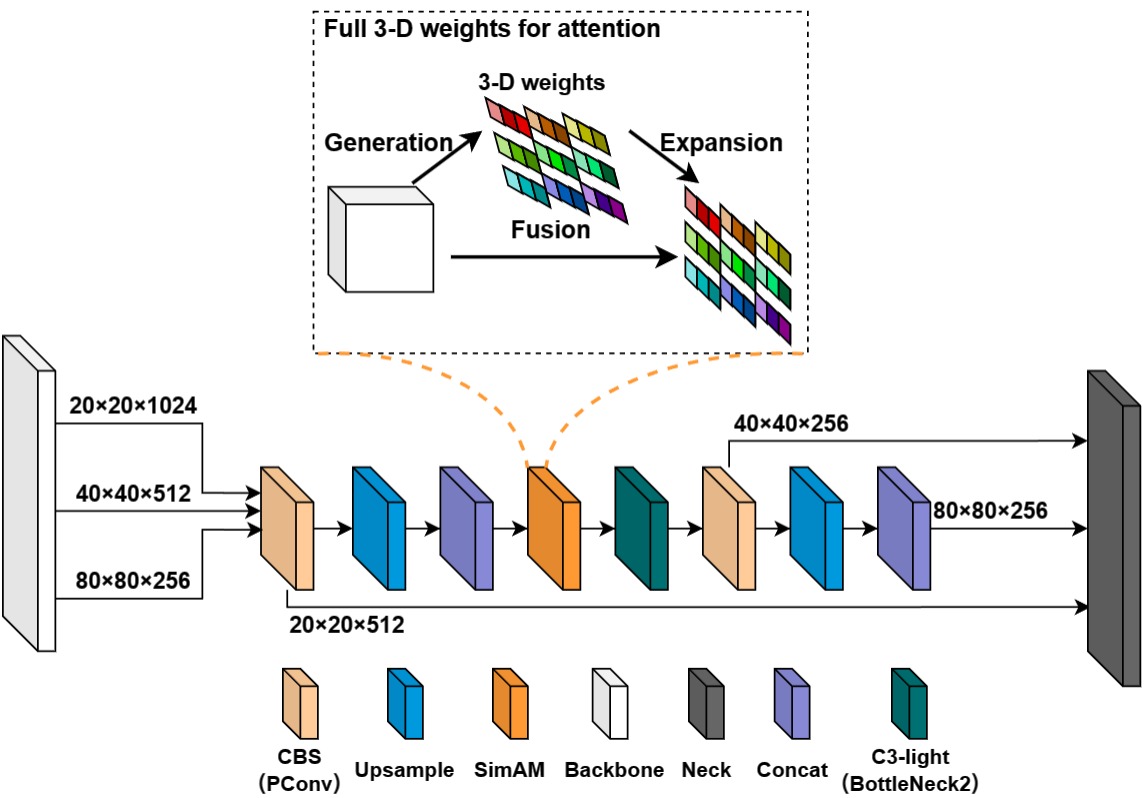
Feature fusion network with attention mechanism.

The SimAM module is better than conventional 1D and 2D weight attention. It adds no parameters to the original neural network and deduces the input information in the convolutional layer to obtain the 3D attention weight of the features. The feature fusion network with a coordinate attention mechanism is shown in Figure 13. The input image is 640 × 640 × 3. The backbone layer of the network model extracts feature information at different scales—80 × 80 × 256, 40 × 40 × 512, 20 × 20 × 1024—and performs multi-scale fusion through the neck layer. The SimAM module is introduced to obtain sufficient feature information. The outputs are 80 × 80 × 256, 40 × 40 × 256, and 20 × 20 × 512 into the next layers. The SimAM accelerates the model’s convergence and further improves the detection performance of the model.

### 3.4. Evaluation Metrics

We used precision, recall, F1-score, average precision(AP), and mean average precision (mAP) metrics to characterize the performance of models [47]. The metrics are shown in the Equations (1)–(5), where TP (True Positive) means that categorized positive class and is predicted to be positive class, FP (False Positive) denotes that the negative class is predicted to be positive class, and FN (False Negative) indicates that the positive class is predicted to be negative class.

Precision, which represents the proportion of cases that are categorized as positive and are actually positive throughout the example, is calculated via Equation (1):

(1) $$Precision=\frac{TP}{TP+FP}.$$

Recall, which indicates the proportion of actual positive cases that are predicted to be positive, is calculated via Equation (2):

(2) $$Recall=\frac{TP}{TP+FN}.$$

The F1 score is the reconciled mean of precision and recall, with 1 being the best and 0 being the worst, and is calculated via Equation (3):

(3) $$F1=2\times \frac{Recall\times Precision}{Recall+Precision}.$$

AP (average precision) denotes the area under the precision–recall curve and the closed curve consisting of the coordinate axes, and it is calculated via Equation (4):

(4) $$AP=\int_{0}^{1}\left(Precision\times Recall\right)\;dx.$$

mAP${}_{0.5:0.95}$ is the average mAP computed over ten different IoU thresholds (from 0.5 to 0.95 in steps of 0.05). In this experiment there is only one category of “apple”, so the mAP is equal to AP. mAP${}_{0.5:0.95}$ is calculated via Equation (5):

(5) $$mAP=\frac{1}{N}\sum_{i=1}^{N}AP_{i}.$$

### 3.5. Experiment and Model Training

We first verify the effectiveness and efficiency of our proposed YOLO-CS compared with the original YOLOv5n; then, we evaluate YOLOv5-CS over state-of-the-art detection models, including both two-stage and one-stage models on our apple dataset. Finally, we conduct a brief ablation. For fair comparisons, all networks were implemented on pytorch with the same settings in our computer. The hardware configuration is Intel(R) Core(TM) i7 4790k 4.40 GHz CPU, GPU is NVIDIA GeForce GTX 1060 with 6 GB of video memory, the memory size is 24 GB, the Python version is 3.9.7, and the CUDA driver version is 11.7.

**Figure 14:**
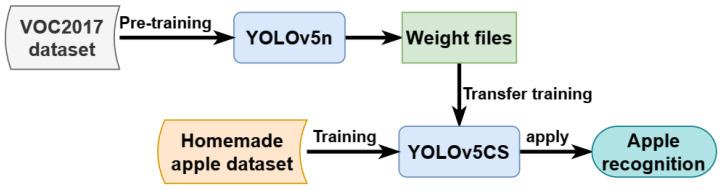
Transfer training process.

We initialized the model on the COCO2017 dataset with a pre-trained model weight file, which is then used for training on our apple dataset. The training process is shown in Figure 14. Transfer learning can shorten the training time, accelerate the convergence of the network, and improve the training performance. The parameters of the trained YOLOv5n and YOLOv5-CS models are given in Table 8.

In detail, the input image were cropped to 640 × 640 from the original images during training. We trained the dataset with a batch size of 12 and training epochs of 200. The learning rate started from 0.01 with a cosine-annealing schedule to adjust the learning rate at every epoch. Weight decay was set to 0.0001. We used the K-means clustering algorithm to generate anchor boxes according to the size of dataset and proportion of the true bounding boxes.

## 4. Discussion

(1) We propose a real-time lightweight model for apple detection. Compared with the YOLO family (i.e., YOLOv8, YOLOv7, YOLOv5l), the YOLOv5n network is simpler and fastery, and the AP is over 75%. We adopt YOLOv5n as the base model to meet the requirements of model size and detection speed and then improve the model accuracy.
(2) The PConv of C3-light efficiently extracts spatial features. It leverages redundancy within the feature maps and performs a convolution only on a subset of the input channels, leaving the remainder intact. The C3-light module accelerates the convergence of the model by reducing computational redundancy and memory access structure. Furthermore, it reduces model size and boots the running speed.
(3) The comparatively worse performance of YOLOv5n is mainly due to its deficit in detecting small apple objects under instances of fruit overlap, leaves occlusions, and illumination variations. SimAM is a simple but very effective attention module for convolutional neural networks, which effectively improves the model’s sensitivity to small and dense apple targets then improves the detection accuracy of apple detection tasks in complex natural orchards.
(4) The YOLOv5-CS model correctly detects the most significant number of apple objects and guarantees accuracy and detection speed, making it easier to deploy on embedded platforms. In addition, YOLOv5-CS has the lowest false detection rate, which avoids erroneous manipulations of the robot arm due to false detection during apple picking and improves the overall picking efficiency of the robot. In summary, the YOLOv5-CS model achieves a trade-off between model weight and detection accuracy, improving detection speed while ensuring a low false detection rate which is suitable for automatic apple picking.

## 5. Conclusions

We introduced the lightweight C3-light module to replace the C3 module in the feature extraction layer to improve the lightweight network architecture. We then integrated the parameter-free attention coordinate SimAM into the feature fusion layer to improve the model detection accuracy and proposed the YOLOv5-CS network model. YOLOv5-CS runs in 14.1 ms at 99.10% AP and is much smaller and more accurate. We compared the YOLOv5-CS network with mainstream object detection models, and extensive experimental analysis shows that the YOLOv5-CS outperforms other networks in terms of AP, speed, and model size, which aligns with the deployment and application of agricultural embedded devices. The proposed YOLOv5-CS model guarantees detection accuracy and speed while also meeting the real-time requirements of the picking robot.

However, YOLOv5-CS only works on mature apples and has yet to be applied on embedded platforms. In the future, we will further optimize the detection model, test the efficiency of the automated device-loading algorithm, and evaluate the effectiveness and practicality of the model in natural apple orchard environments.

## Figures and Tables

**Table 1:** Comparison of YOLOv5-CS vs. YOLOv5n.

Model	Precision/%	Recall/%	F1/%	AP/%	mAP${}_{0.5:0.95}$/%	GFLOPs	Model Size/MB	Inference Time/ms
YOLOv5n	85.92	85.77	85.80	75.98	61.83	4.5	7.6	12.7
YOLOv5n-CS	97.81	97.32	97.55	99.10	70.25	3.8	6.25	14.1

**Table 2:** Comparison of accuracy with different models.

Model	Precision/%	Recall/%	F1/%	AP/%	mAP${}_{0.5:0.95}/\%$	Model Size/MB	GFLOPs/%	Inference Speed/ms
Faster R-CNN	78.55	81.75	85.00	80.63	-	528	15.5	900
RetinaNet	85.55	84.84	86.00	86.10	-	98	3.9	1100
YOLOv5n	85.92	85.77	85.80	75.98	61.83	7.5	4.5	12.7
YOLOv5s	88.52	88.49	89.37	79.86	63.81	28.6	16.5	15.7
YOLOv5m	95.49	94.62	96.14	86.86	70.49	84.6	49	20.11
YOLOv5l	97.82	96.15	98.34	90.43	72.43	186.0	109	24.77
YOLOv7	96.77	96.02	96.39	88.31	70.89	72.1	104.7	21.3
YOLOv8	99.2	98.1	98.65	99.40	77.8	83.7	165.2	35.4
YOLOv5-CS	97.81	97.32	97.55	99.10	70.25	6.25	3.8	14.1

**Table 3:** Parameters of the C3 and C3-light modules.

Module	GFLOPs	Parameters
C3	2.46	972,096
C3-light	1.67	664,464

**Table 4:** Performance of lightweight network model.

Model	C3-Light	SimAM	AP%	GFLOPs	Parameters
YOLOv5n	×	×	75.98	4.5	1,872,157
YOLOv5-C	✓	×	70.63	3.2	1,564,525
YOLOv5-CS	✓	✓	99.10	3.8	1,564,525

**Table 5:** Data augmentation methods.

Augmentation	Main Function	Parameter
Gaussian blur	GaussianBlur( )	(0.5, 3.0)
Average blur	AverageBlur( )	(2, 11)
Colorspace adjustment	WithColorspace( )	(10, 50)
Brightness adjustment	WithBrightnessChannels( )	(−50, 50)
Gaussian noise	GaussianNoise( )	3
Horizontal flip	Fliplr( )	1
Vertical flip	Flipud( )	1
Image zoom	Resize( )	(0.5, 0.8)

**Table 6:** Distribution of apple dataset.

Category	Original	Augmentation	Training Set	Validation Set	Test Set
apple image	500	2700	2160	270	270
“apple” label	7046	36,912	29,600	3691	3621

**Table 7:** Parameters comparison of YOLOv5 family.

Model	Depth	Width	Model Size/MB
YOLOv5l	1.00	1.00	186.0
YOLOv5m	0.67	0.75	84.6
YOLOv5s	0.33	0.5	28.6
YOLOv5n	0.33	0.25	7.5

**Table 8:** Training parameters used in network.

Training Parameters	YOLOv5n	YOLOv5-CS
Initial learning rate	0.01	0.01
learning rate	0.0001	0.0001
Batch size	12	12
Epochs	200	200
Train crop size	640 × 640	640 × 640
Class	1	1
IoU Threshold	0.5	0.5
Confidence Threshold	0.25	0.25

## Data Availability

Not applicable.
